# Entropy Applications in Environmental and Water Engineering

**DOI:** 10.3390/e20080598

**Published:** 2018-08-10

**Authors:** Huijuan Cui, Bellie Sivakumar, Vijay P. Singh

**Affiliations:** 1 Key Laboratory of Land Surface Pattern and Simulation, Institute of Geographic Sciences and Natural Resources Research, Chinese Academy of Sciences, Beijing 100101, China; 2 UNSW Water Research Centre, School of Civil and Environmental Engineering, The University of New South Wales, Sydney, NSW 2052, Australia; 3 Department of Civil Engineering, Indian Institute of Technology Bombay, Powai, Mumbai, Maharashtra 400076, India; 4 State Key Laboratory of Hydroscience and Engineering, Tsinghua University, Beijing 100084, China; 5 Department of Biological and Agricultural Engineering & Zachry Department of Civil Engineering, Texas A and M University, College Station, TX 77843-2117, USA

**Keywords:** entropy theory, complex systems, hydraulics, hydrology, water engineering, environmental engineering

Entropy theory originated from the second law of thermodynamics, and its extension to information theory became a versatile tool for modeling complex systems and associated problems. Entropy has found applications in a wide range of problems in earth, environmental, and geographical sciences. This special issue focuses on the applications of entropy theory in environmental and water engineering.

Entropy is considered as a measure of uncertainty or the amount of information gained through measurements of a random variable. Baran et al. [1] defined entropy as an invariant measure function and extended the assessment of uncertainty. They stated that entropy did not mean an absolute measure of information, but as a measure of the variation of information, which intends to help solve information-related problems in hydrologic monitoring.

Based on the review of entropy modeling in water engineering by Singh [2], applications of entropy theory can be classified into three groups: (1) statistical or empirical, (2) physical, and (3) mixed. The first group focuses on probability determination and requires entropy maximization, including frequency analysis, parameter estimation, network evaluation and design, spatial and inverse spatial analysis, flow forecasting, and complexity analysis, and clustering. The second group involves deriving physical relations either in time or in space, such as rainfall-runoff modeling, infiltration, soil moisture, velocity distribution, and flow duration curve, among others. For instance, Zhang et al. [3] showed how the entropy parameter derived from the entropy-based flow duration curve is linked to the drainage area, impacted by reservoir operation, and possibly climate change. The third group is a mixture of the above two and includes applications such as the reliability of water distribution systems. Numerous examples of these can be found in the review article on the Tsallis entropy, by Singh et al. [4].

Applications in the first category are common in hydrology, and many studies in this special issue belong to this category. Using the principle of maximum entropy (POME), the four-parameter exponential gamma distribution, generalized gamma distribution, and generalized beta distribution were derived for flood frequency analysis in [5,6,7]. Chen et al. [8] showed that entropy-based generalized distributions can further be used for the analysis of extreme rainfall with Bayesian technique.

Keum et al. [9] reviewed applications of entropy in water monitoring network design, including precipitation, streamflow and water level, water quality, soil moisture, and groundwater network. The network designed by Yeh et al. [10] showed how to optimize the rainfall network with both radar and entropy. Santonastaso et al. [11] introduced flow entropy as a measure of network redundancy and a proxy of reliability in optimal network design procedures, which can identify the tradeoff between network cost and robustness. Besides water distribution networks, entropy was used to develop an integrated optimization model for the spatial optimization of agricultural land use based on crop suitability, spatial distribution of population density, and agricultural land use data [12]. Similar to optimization, entropy was also used to determine weights of evaluating indicators in a fuzzy system [13,14] and can be applied in combined forecasting of rainfall [15]. 

Applications in this special issue have used several different entropy formulations, such as the Shannon, Tsallis, Rényi, Burg, Kolmogorov, Kapur, configurational, and relative entropies, which can be derived in time, space or frequency domain. The sample entropy was used to investigate streamflow and water level complexity of the Poyang Lake over multiple time-scales [16]. The connection entropy was applied to establish a water resources vulnerability framework [17]. The generalized space q-entropy was employed for spatial scaling and complexity properties of Amazonian radar rainfall fields [18]. The Kolmogorov complexity and the Shannon entropy were combined to evaluate the randomness of turbulence [19]. Cheng et al. [20] employed several entropy measures, such as intensity entropy, apportionment entropy, and marginal entropy to investigate spatial and temporal precipitation variability. Defined in frequency or spectral power domain, entropy can be used for spectral analysis. In this way, entropy can be used in time series analysis and forecasting and, hence, for characterizing stochastic and periodic patterns [21].

Mutual information is a measure of mutual dependence between two variables and can be determined from marginal and joint entropies. It is an efficient tool to investigate linear or non-linear interactions, such as the relationship between vegetation pattern and hydro-meteorological elements [22], the relationship between annual streamflow, extreme precipitation and ENSO (El Niño–Southern Oscillation) [23], and the relationship between soil water content and its influencing factors [24]. 

More recently, entropy-based concepts have been coupled with other theories, including copula, wavelets, and ensemble filter, to study various issues associated with environmental and water resources systems. Guo et al. [25] developed a coupled maximum entropy-copula method for hydrologic risk analysis through deriving bivariate return periods, risk, reliability, and bivariate design events, which shows that the maximum entropy theory is beneficial for improving the performance of copulas. As a result, the distribution derived by the maximum entropy-copula model outperforms the conventional distributions for the probabilistic modeling of floods and extreme precipitation events. Foroozand et al. [26] combined entropy with ensemble filter method to evaluate model performance, which can mitigate the computational cost of the bootstrap aggregating method. The entropy concept was linked to the notion of elasticity to assess catchment resilience, which determined the changes in mean annual runoff [27].

Other than the above probabilistic entropy, the classical thermodynamic entropy concept was also visited in the special issue, by Koutsoyiannis [28], in the entropy production. Entropy production was explored within stochastics in logarithmic time, related to model identification and empirical fitting, which was applied to an extraordinarily long time series of turbulent velocity and showed how a parsimonious stochastic model can be identified and fitted.

The above contributions to this special issue show the enormous scope and potential of entropy theory in advancing research in the field of environmental and water engineering, including establishing and explaining physical connections between theory and reality.
